# Lower urinary tract signs and symptoms in patients with COVID-19

**DOI:** 10.1186/s12879-021-06394-z

**Published:** 2021-07-26

**Authors:** Aida Javan Balegh Marand, Christian Bach, Dick Janssen, John Heesakkers, Morteza Ghojazadeh, Thomas Alexander Vögeli, Hanieh Salehi-Pourmehr, Hadi Mostafae, Sakineh Hajebrahimi, Mohammad Sajjad Rahnama’i

**Affiliations:** 1 Uniklinik RWTH Aachen, Pauwelsstrasse 30, 52074 Aachen, Germany; 2 Radboud University, Nijmegen, The Netherlands; 3 Society of Urological Research and Education (SURE), Heerlen, The Netherlands; 4 Research Center for Evidence-Based Medicine, Tabriz University of Medical Sciences, Tabriz, Iran; 5 Medical University of Vienna, Tabriz, Austria

**Keywords:** Lower urinary tract symptoms (LUTS), COVID-19, SARS-CoV-2, Hematuria, Urine, leukocyturia

## Abstract

**Background:**

The type of pneumonia that is caused by the new coronavirus (SARS-CoV-2) has spread across the world in a pandemic. It is not clear if COVID-19 patients have any lower urinary tract signs or symptoms.

**Methods:**

The effect of COVID-19 on lower urinary tract function was studied in a prospective multi-centre, observational study including 238 patients who were admitted with symptoms caused by COVID-19 to the university hospital of Aachen in Germany and Tabriz in Iran.

**Results:**

None of the patients reported to have any lower urinary tract symptoms. SARS-CoV-2 was found in the urine of 19% of the tested patients.

The mortality rate in COVID-19 infected patients with microscopic haematuria together with white blood cells in their urine, was significantly increased from 48 to 61% in the Tabriz cohort (*p*-value = 0.03) and from 30 to 35% in the Aachen cohort (p-value =0.045). Furthermore, in the group of patients with SARS-CoV-2 urine PCR, the mortality rate rose from 30 to 58%. (*p*-value =0.039).

**Conclusion:**

Patients admitted with COVID-19 did not report to have any lower urinary tract symptoms, even those patient who had a positive Urine SARS-CoV-2 PCR.

In addition, hematuria, WBC in urine as well as SARS- CoV-2 presence in urine, were found to be strong negative prognostic factors in admitted COVID-19 patients.

## Background

The latest and most momentous threat to our world is the pandemic caused by the new coronavirus (SARS-CoV-2). Priorities in medical and surgical healthcare have shifted drastically due to this pandemic. All fields and specialties are facing new challenges and urology is no exception.

The 2019 novel Coronavirus (2019-nCOV) was isolated on the 7th of January by Chinese scientists who then commenced genome sequencing which was later provided to the World Health Organization (WHO) on January 12th 2020 [1].

The WHO revealed the causative virus as Severe Acute Respiratory Syndrome Coronavirus 2 (SARS-CoV-2) and the pneumonia as Coronavirus Disease 2019 (COVID-19). COVID-19 was declared a pandemic on March 11th 2020 by the WHO [1].

Chest computed tomography (CT) and plain X-ray play a crucial role in the detection of early pulmonary changes and surveillance of patients with COVID-19 [2, 3].

Although respiratory symptoms are the predominant presentation of COVID-19 among symptomatic infected patients, it is important to emphasize that multiple organ involvement including the gastrointestinal tract, central nervous system, cardiovascular system, liver, bone marrow and kidney have already been reported in patients infected with SARS-CoV-2 [4, 5]. Besides severe lung failure i.e. Acute Respiratory Distress Syndrome (ARDS), heart and kidney failure have been recorded among infected patients [6].

Hematuria associated with SARS-CoV-2 infection has also been reported in both adults and children [5, 7].

A patient’s infectivity is determined by the presence of the virus in different body fluids, secretions, and excreta. The persistence and clearance of viral RNA from different specimens of patients with 2019 novel coronavirus disease (COVID-19) remained unclear. However, in both human and animal studies, limited persistence of SARS-COV-2 in urine has been detected [8, 9].

To date, there have been no studies published on the effect of SARS- CoV-2 infection on the lower urinary tract function.

In this study we aimed to investigate the effect of SARS- CoV-2 infection on the lower urinary tract function in admitted COVID-19 patients.

## Methods

Between February and June 2020, all patients who were admitted to the two study hospitals (Aachen University Hospital in Germany and Tabriz University Hospital in Iran), and who tested positive for SARS-CoV-2 were included in our study.

In this prospective multi-centre, observational study, the blood and urine data where collected by the treating medical staff and entered to the digital medical system.

For pathogen diagnosis (viral, bacterial, fungal), bronchoscopy and broncho-alveolar lavage with a total volume of 160 ml was performed in all intubated patients. In not intubated patients, real-time polymerase chain reaction (PCR) was conducted from deep nasal and pharyngeal swaps.

For SARS-CoV-2 detection the viral load was recorded by PCR of respiratory material and in some cases in urine. Threshold cycles for the S-gene < 20 were classified as high. Values > 30 were considered low viral load; values between 20 and 30 were classified as moderate viral load.

Serum, urine, and stool were also tested for SARS-CoV-2. Urine parameters were recorded over time, such as erythrocyte count (RBC), white blood cell count (WBC) and proteinuria. Descriptive and analytical statistics including chi-square test and Fisher’s Exact test was employed using IBM SPSS (version 24). All analyses were considered significant when *p* < 0.05.

## Results

The study population was made up of two cohorts of COVID-19 patients who were hospitalized in the University Hospitals Aachen in Germany and Tabriz in Iran. A total of 238 patients were included in our study. The two cohorts existed of: 133 patients (41 females - 92 males) who were admitted at the University Hospital in Aachen, Germany and 105 patients (39 females and 66 males) who were admitted at the University Hospital of Tabriz in Iran. The patients’ data and characteristics are listed in Table 1.

**Table 1:** Patient characteristics and data of the two cohorts

City	Tabriz (***N***^a^ = 105)	Aachen (***N***^a^ = 133)	***P-***Value
Variables			
**Age in years (Range)**	60 (16–89)	65 (25–88)	*0.18*
**Sex**			
**Female**	39 (37%)	41 (30%)	*0.28*
**Male**	66 (63%)	92 (70%)	
**Bacterial Urine Culture positive**	*N* = 19 2 positive (11%)	*N* = 103 48 positive (47%)	*0.003*
**Urine SARS-CoV-2 PCR**	None	*N* = 63 12 positive (19%)	*–*
**Urine Red blood cell count** (RBCs/μl < 25.0)	*N* = 99 67 positive (68%)	*N* = 82 64 positive (76%)	*0.12*
**Urine White blood cell count** (WBCs/μl < 20.0)	*N* = 99 65 positive (66%)	*N* = 82 34 positive (42%)	*0.001*
**Blood Urea (mg/dL)** Normal values(16-48 mg/dL)	*N* = 92 43 pts. elevated (47%)	*N* = 133 90 pts. elevated (68%)	*0.002*
**Mortality**	50 (48%) (14 women, 36 men)	40 (30%) (13 women, 27 men)	*0.005*

^a^**N. is the total number of the included COVID-19 patients who had an analysis of each mentioned parameter**

The average age of all patients studied was 65 years. In the Tabriz-cohort, the average age was 60 years (range 16–89) and in the Aachen-cohort it was 65 years (25–88).

The main primary symptoms that were reported were: fever, shortage of breath and coughing. None of the studied patients reported to have any lower urinary tract symptoms.

The average time between the occurrence of symptoms and hospitalization in both cohorts was four days.

From all admitted patients, 90 patients passed away: 40 in Aachen (27 male, 13 female) and 50 in Tabriz (36 male, 14 female).

From the included 238 COVID-19 infected patients, 181 patients had a urinalysis of whom, 131 patients had microscopic hematuria (72%) and 99 patients had white blood cells in their urine (54%), (Table 1).

The mortality rate in COVID-19 infected patients with microscopic hematuria together with white blood cells in their urine, was significantly increased from 48 to 61% in the Tabriz cohort (*p*-value = 0.03) and from 30 to 35% in the Aachen cohort (p-value =0.045), as shown in Table 2. In addition, the urine of 63 patients admitted in Aachen, was checked for SARS-CoV-2 presence by PCR. From these patients, 51 were negative (81%) of whom 13 patients died (25,5%). Moreover, 12 patients tested positive for SARS-CoV-2 in their urine (19%). From these 12 patients, 7 patients died (58%). This means that, in the group of patients with SARS-CoV-2 urine PCR, the mortality rate rose from 30 to 58%. (*p*-value =0.039) (Table 2).

**Table 2:** Comparison of mortality according to different urine findings of COVID-19 Patients

Urine-Test	Tabriz	Aachen	Mortality Tabriz	Mortality Aachen
	*N* = 105 99 (94%)	*N* = 133 82 (62%)	*N* = 99 47 (48%)	*N* = 82 27 (33%)
WBC	*N* = 99 **65 (66%)**	*N* = 82 **34 (41%)**	*N* = 65 **35 (54%)**	*N* = 34 **11 (32%)**
RBC	*N* = 99 **67 (68%)**	*N* = 82 **64 (78%)**	*N* = 67 **40 (60%)**	*N* = 64 **23 (36%)**
WBC + RBC	*N* = 99 **54 (54%)**	*N* = 82 **29 (35%)**	*N* = 54 **33 (61%)**	*N* = 29 **10 (35%)**
Positive Urine COVID PCR test	–	*N* = 63 **12 (19%)**	–	*N* = 12 **7 (58%)**

*RBC* Red blood cell count, *WBC* White blood cell count

From the total of 238 patients included in our study, 122 patients had a bacterial urine culture of whom 50 patients (41%) had a positive bacterial urine culture. The most common uropathogens were (*E. coli*, *Klebsiella pneumoniae* and Proteus). The mortality among the patients with a positive bacterial urine culture was 26% (13 patients).

## Discussion

The diagnosis of COVID-19 can be challenging as patients often present with unclear or even subclinical signs of disease [10]. In our study, none of the COVID-19 patients reported any LUTS or had urinary retention or incontinence. Even in those 12 patients who tested positive for viral RNA in their urine. A study that focused on an increase in urinary frequency as a symptom of COVID-19 identified this in seven males out of 57 patient admitted with COVID-19 [7]. Interestingly, no viral RNA was found in the urine of these patients [7].

Regarding the urine analysis of COVID-19 patients in our study, we can state that the presence of WBC and RBC in the urine as well as the presence of COVID-19 virus in urine, seems directly related to an increase of mortality in these patients (Table 2). In patients with microscopic hematuria together with white blood cells in their urine, the mortality was significantly increased from 48 to 61% in the Tabriz cohort (*p*-value = 0.03) and from 30 to 35% in the Aachen cohort (*p*-value =0.045). In addition, Moreover, in the group of patients with SARS-CoV-2 urine PCR, the mortality rate rose from 30 to 58%. (*p*-value =0.039).

In our series, the two cohorts of Tabriz and Aachen, were not statistically different regarding age and male-female ratio (p-value 0,18 and 0,28).

The mortality rate in Tabriz (48%) was significantly higher than in Aachen 30% (p-value 0.005). This might be explained by many factors that were not investigated in our study such as the availability of resources as well as patient comorbidities.

There were significantly less patients who had a urine culture in the Tabriz cohort which would explain the differences in the positive culture rates.

Microscopic haematuria was seen in a majority of tested COVID-19 patients of Tabriz and Aachen (68 and 78%, respectively) and was not due to a positive bacterial urine culture in most cases (Table 1).

Hematuria has been reported in other viral respiratory infections including influenza A and B as well as adenovirus [11]. Moreover, kidney injury in hospitalized patients with COVID-19 appears to be a frequent finding [12]. Different symptoms have been reported ranging from, from mild hematuria to severe renal failure [11]. The underlaying pathophysiology is not well known. However, hypotheses have been put forward on the cytopathic effects of the virus as well as the immune-complexes mediated damage [11]. Furthermore, indirect effects on renal tissue, including hypoxia, and rhabdomyolysis due to the cytokine inflammatory response to the virus might play a role as well.

In our study, we have found increase white blood cells (WBC) in the urine of about half of the tested COVID-19 patients (Table 2). In most of the cases there was a negative bacterial urine culture.

The time from suspected exposure to the onset of COVID-19 and worsening hematuria ranged between 5 and 8 days in the reported cases.

In the face of a possible increase and new waves of the pandemic, clinicians should be aware that a non-typical course of hematuria or increased WBC in urine might be related to a SARS- CoV-2 infection, especially in patients with a preexisting condition of the urinary tract [12].

It is known that the mortality rates are high between the COVID-19 patients with acute kidney injury (60–90%) [13].

Viral RNA has been identified in the urine samples of COVID-19 patients even after recovery from respiratory symptoms [8]. In our study, SARS-CoV-2 was found in the urine of 19% of the tested patients.

Angiotensin-converting enzyme 2 (ACE2), the receptor for SARS-CoV-2 and responsible for host cell entry might be a another possible link to multi organ failure of the COVID-19 patients. ACE2 is expressed different organs such as the hearth, the gastrointestinal tract, bone marrow and kidneys and bladder [14].

**Fig. 1:**
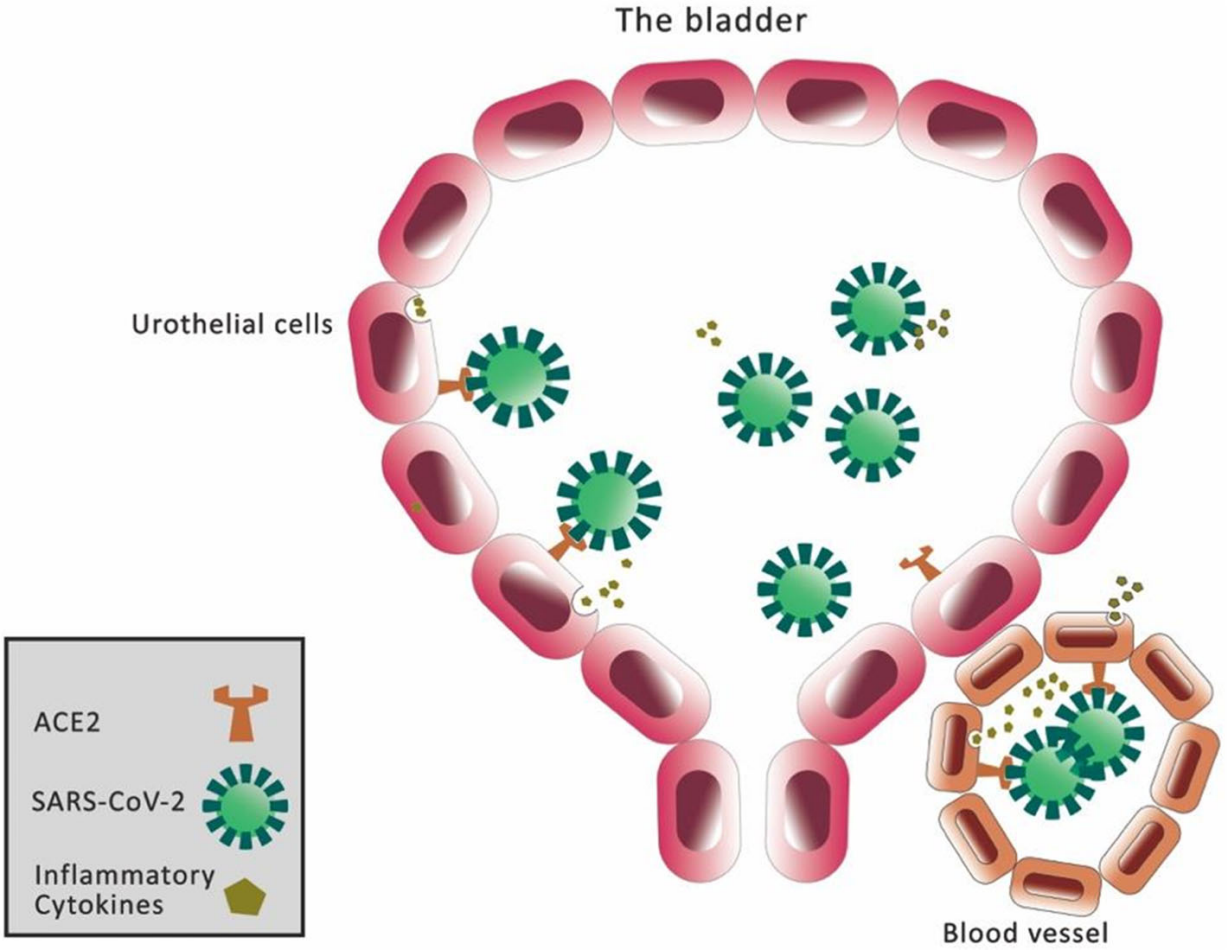
A cartoon of SARS-CoV-2 Infection in the Urinary Bladder produced by the authors

Recent studies revealed the cell-surface protein angiotensin-converting enzyme 2 (ACE2) as the main receptor for the SARS-CoV-2 spike protein [15]. Investigations of the distribution across many different tissues revealed that ACE2 expression was highest in lung, intestines, and kidney, but it was also high in 2.4% of urothelial cells, which might be a link with lower urinary tract symptoms [16]. In Next to the kidney tissue bladder urothelium has cells that express ACE2 [12, 16, 17]. We hypothesize a possible link between the lower urinary system and SARS-CoV-2 through the ACE2 (Fig. 1).

Our study is limited by the relatively small sample size and the emergency setting in which most patients were included in the study. More future studies with larger sample size are needed to determine the exact effect of SARS- CoV-2 infections on the lower urinary tract.

## Conclusion

We found no lower urinary tract symptoms in patients admitted with COVID-19.

Even the presence of SARS- CoV-2 did not cause any urinary symptoms. It can be concluded that hematuria, WBC in urine as well as SARS- CoV-2 positivity in urine are negative prognostic factors in admitted COVID-19 patients.

## Data Availability

The datasets used and/or analysed during the current study are available from the corresponding author on reasonable request.

## Abbreviations

SARS-CoV-2: Severe Acute Respiratory Syndrome Coronavirus 2
COVID-19: Coronavirus Disease 2019
ACE2: Angiotensin-converting enzyme 2
WBC: white blood cells
RBC: Erythrocyte count
WHO: World Health Organization
